# Characterization and Evolution of microRNA Genes Derived from Repetitive Elements and Duplication Events in Plants

**DOI:** 10.1371/journal.pone.0034092

**Published:** 2012-04-16

**Authors:** Jie Sun, Meng Zhou, Zhitao Mao, Chuanxing Li

**Affiliations:** College of Bioinformatics Science and Technology, Harbin Medical University, Harbin, China; Michigan State University, United States of America

## Abstract

MicroRNAs (miRNAs) are a major class of small non-coding RNAs that act as negative regulators at the post-transcriptional level in animals and plants. In this study, all known miRNAs in four plant species (*Arabidopsis thaliana*, *Populus trichocarpa*, *Oryza sativa* and *Sorghum bicolor*) have been analyzed, using a combination of computational and comparative genomic approaches, to systematically identify and characterize the miRNAs that were derived from repetitive elements and duplication events. The study provides a complete mapping, at the genome scale, of all the miRNAs found on repetitive elements in the four test plant species. Significant differences between repetitive element-related miRNAs and non-repeat-derived miRNAs were observed for many characteristics, including their location in protein-coding and intergenic regions in genomes, their conservation in plant species, sequence length of their hairpin precursors, base composition of their hairpin precursors and the minimum free energy of their hairpin structures. Further analysis showed that a considerable number of miRNA families in the four test plant species arose from either tandem duplication events, segmental duplication events or a combination of the two. However, comparative analysis suggested that the contribution made by these two duplication events differed greatly between the perennial tree species tested and the other three annual species. The expansion of miRNA families in *A. thaliana*, *O. sativa* and *S. bicolo*r are more likely to occur as a result of tandem duplication events than from segmental duplications. In contrast, genomic segmental duplications contributed significantly more to the expansion of miRNA families in *P. trichocarpa* than did tandem duplication events. Taken together, this study has successfully characterized miRNAs derived from repetitive elements and duplication events at the genome scale and provides comprehensive knowledge and deeper insight into the origins and evolution of miRNAs in plants.

## Introduction

MicroRNAs (miRNAs) are a major class of small non-coding RNAs that act as negative regulators at the post-transcriptional level in animals and plants [1], [2]. The origins and evolution of miRNAs have been a very important area of research in miRNAomics and have attracted much interest since the discovery of the miRNAs, *lin-4* and *let-7*, in *Caenorhabditis elegans*. A large number of studies have contributed to the origins and evolution of miRNAs in animals and plants. Several hypotheses have been proposed to explain the origins and family expansion of miRNAs, including random hairpin sequences [3], [4], repetitive elements [5], [6], [7], [8], genomic duplication events (tandem duplications and segmental duplications) [8], [9], [10] and inverted duplication of target genes [11].

It is well known that repetitive elements are one of the major components of most plant nuclear genomes, constituting ∼10%, ∼40%, ∼35% and ∼61% of the *Arabidopsis thaliana*, *Populus trichocarpa*, *Oryza sativa* and *Sorghum bicolor* genomes, respectively [12], [13], [14], [15]. Accumulating evidence from many studies in animals has shown that repetitive elements, especially transposable elements (TEs), have contributed to the formation of miRNAs. Recent studies have identified a large number of repeats-derived miRNAs in the human, rhesus and mouse genomes [8]. In plants, such as *A. thaliana* and *O. sativa*, some miRNAs were initially found to be co-located with TE sequences [16]. Another study by Li *et al.* revealed that 106 miRNAs were homologous to TEs in three plant species (*A. thaliana* **,** *O. sativa* and *T. aestivum*) [7]. However, there is very little known in plants about the characteristics and evolutionary patterns of miRNAs derived from repetitive elements distinguishable from non-repeat-related miRNAs in plants.

Previous studies have shown that duplication events play an important role in gene duplication [17], [18], and may also contribute to the origins and expansion of miRNA families [8], [9], [10]. Tandem duplications can result in paralogous miRNA sequences that are located on the same transcript and organized as tandem paralog clusters [10], [19]. Segmental duplications are physically interspersed blocks of duplicated material in a genome and, in a recent study, have been proven to prompt the expansion of some miRNA families in mammalian genomes [8]. In plants, the evolution of miRNAs through tandem or segmental duplication events was reported in several early studies. However, these were performed only on the relatively small number of miRNA genes and were limited to *A. thaliana* and *O. sativa* [9], [20].

An increasing number of plant miRNA genes have been discovered through experimental and bioinformatic studies in plants [21], and they provide an opportunity to study the origins and evolution of miRNAs. In this study, a more extensive analysis, at the genome scale, was performed in order to provide a complete mapping of all the miRNAs on repetitive elements in the four test plant species. In addition, miRNAs derived from repetitive elements were further characterized by undertaking a series of studies to compare various features between the repeats-related miRNAs and non-repeats-related miRNAs. Then a combination of computational and comparative genomic approaches was used to systematically identify and characterize the miRNAs that had originated from, or whose family had been expanded by, tandem or segmental duplication events in the four test plants.

## Results

### Identification and evolution of repeats-related plant miRNAs

**Figure 1:**
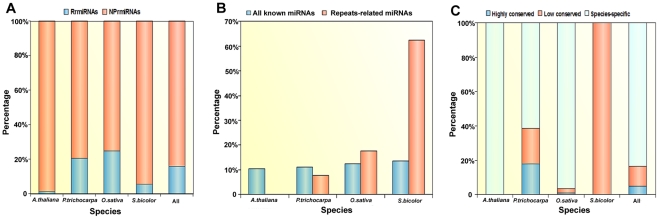
The distribution and conservation of repeat-related miRNAs in the four test plant species. (A) The percentage of RrmiRNAs and NRrmiRNAs in the *A. thaliana*, *P. trichocarpa*, *O. sativa* and *S. bicolor* genomes. (B) The percentage of repetitive element-related miRNAs located in intragenic regions compared to all known miRNAs in the corresponding genome. (C) The percentage of repeat-related miRNAs with differing degrees of conservation.

Previous studies on plant TE-derived miRNAs have reported that TE-derived miRNAs tended to overlap fully or partially with TEs and also shared sequences with TEs [7], [16]. In order to provide a complete mapping of all the miRNAs on repetitive elements in the four test plant species, miRNA hairpin precursor sequences in the four plant species were BLAST searched against the repetitive sequences of corresponding species. A total of 163 miRNAs were identified as repetitive element-related, accounting for 15.9% of all miRNAs in the four test plant species (Fig. 1 and File S1). A recent study on repeats-derived miRNAs in mammalian genomes revealed the distribution of miRNAs that overlapped with repeats in the human, rhesus and mouse genomes [8]. A comparison analysis of plants and animals was performed and found that the percentage of repeats-related miRNAs in plants (15.9%) was significantly lower than in animals (23.5%) (*P*<0.001, Fisher's exact test). It has been reported that the percentage of repetitive element-related miRNAs was very close in different animal species in Yuan's study [8]. In contrast, there are considerable differences in the percentage of repetitive element-related miRNAs between plant species (Fig. 1). Fig. 1 also shows that the percentage of repetitive element-related miRNAs in *P. trichocarpa* and *O. sativa* was significantly higher than the percentage for *A. thaliana* and *S. bicolor* (*P*<0.001, Fisher's exact test).

We next studied the distribution characteristics of the repetitive element-related miRNAs in protein-coding and intergenic regions of the four test plant species. Here, miRNA genes were categorized into two types based on their genomic locations: intragenic miRNAs and intergenic miRNAs. Only miRNAs fully included in UTRs, introns or exons of protein-coding genes were considered as intragenic miRNAs. The results are summarized in Fig 1B. There was no significant difference between species for the percentage of miRNAs located in intragenic regions. However, the repetitive element-related miRNAs show significantly different distributions in both intragenic and intergenic regions compared to all known miRNAs in corresponding species and between different plant species. For *A. thaliana* and *P. trichocarpa* the percentage of repetitive element-related miRNAs located in intragenic regions was lower than for all their miRNAs (Fig. 1B). In contrast to *A. thaliana* and *P. trichocarpa*, a greater percentage of repetitive element-related miRNAs in *O. sativa* and *S. bicolor* were found in intragenic regions compared to the percentage found for all their miRNAs. Inverted-repeat transposable element (MITE) insertions preferentially occurred in genic regions, and these MITEs could be further transformed into RNA hairpins and subsequently form miRNAs [7], [16]. These results might suggest that repetitive element-related miRNAs arose more frequently from intragenic regions in monocots tested than from intragenic regions in eudicots used in this study, indicating that MITE-derived miRNAs were preferentially enriched in monocots compared to in eudicots. This was consistent with the previous studies, which found that many TE-derived miRNAs were encoded by MITEs in rice [7], [16].

To further explore the degree of conservation for repetitive element-related miRNAs, the miRNAs from the four test plant species studied were classified (based on evolutionary conservation across all plant species, as described in the Materials and Methods) into highly conserved miRNAs, low conserved miRNAs and species-specific miRNAs. The results are summarized in Fig. 1C. This study found that in the four test plant species, 83.4% of the repetitive element-related miRNAs were species-specific miRNAs, which was significantly higher than for the low conserved (11.7%) and highly conserved (4.9%) miRNA classes. Piriyapongsa and Jordan also observed that TE-derived miRNAs in *Arabidopsis* and rice had fewer orthologs [16]. Recent studies have suggested that a subset of the predicted lineage-specific miRNAs were associated with transposon-related repeats [22]. Taken together, miRNAs originating from repetitive elements in plants tend to be species-specific or be less evolutionary conserved than non-repeat-derived miRNAs.

### Characterization and differences between RrmiRNAs and NRrmiRNAs

**Figure 2:**
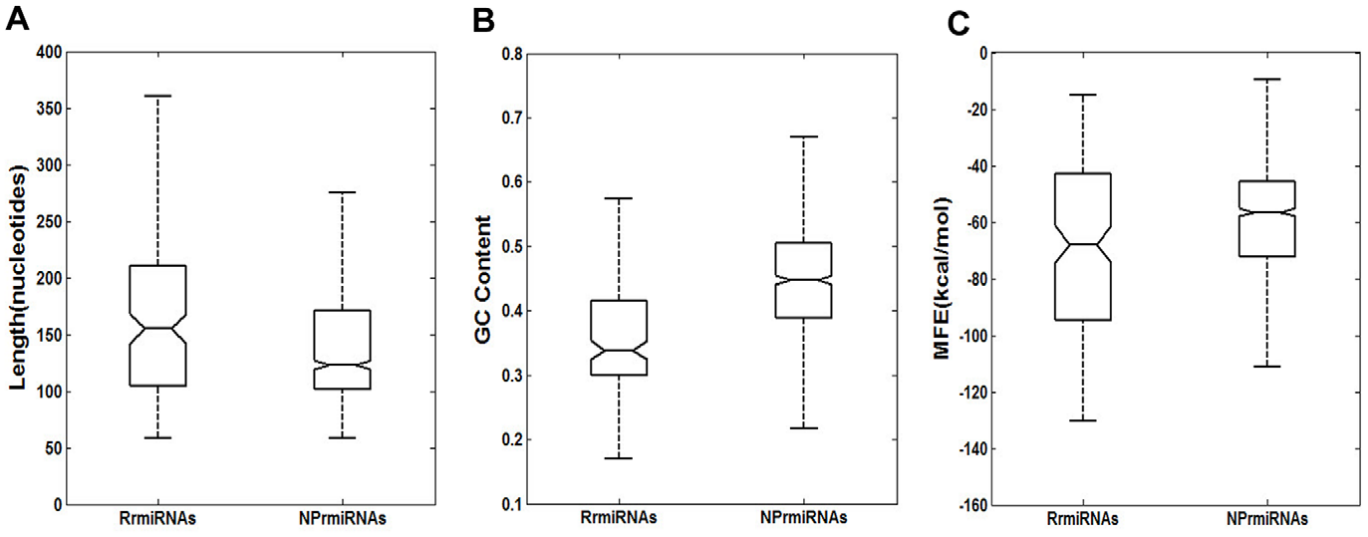
Characterization and variation between RrmiRNAs and NRrmiRNAs. (A) The distribution of miRNA hairpin precursor sequence lengths in RrmiRNAs and NRrmiRNAs. (B) The G-C content in miRNA hairpin precursor sequences in RrmiRNAs and NRrmiRNAs (C) The MFEs for miRNA hairpin precursors in RrmiRNAs and NRrmiRNAs.

To further explore the characterization and differences between RrmiRNAs and NRrmiRNAs, we carried out detailed analysis to examine various characteristics for known miRNAs of four test plant species in the miRBase, including hairpin precursor sequence length, base composition and the minimum free energy (MFE) of hairpin structures. A further comparative analysis between them was also undertaken. In order to examine whether these differences are statistically significant or not, the Wilcoxon rank sum test analysis was performed. First, the hairpin precursor sequence length for RrmiRNAs and NRrmiRNAs in the four test plant species was computed. The results indicated that there was a significant difference between RrmiRNAs and NRrmiRNAs for hairpin precursor sequence length (*p*-value = 1.0623e-05, Wilcoxon rank sum test) (Fig. 2A). The NRrmiRNAs (average 143 bp long) tended to have a shorter sequence than RrmiRNAs (average 165 bp long).

To explore whether the tendency of base composition in hairpin precursor sequence of miRNAs between these two miRNA categories is similar, the base composition pairing was analyzed by measuring the G-C content. There were significant differences in G-C content between RrmiRNAs and NRrmiRNAs (*p*-value = 1.4840e-027, Wilcoxon rank sum test) (Fig. 2B). NRrmiRNAs (average 45.2%) had a higher G-C content than RrmiRNAs (average 36.5%). It is well known that G-C content is important for the stability of RNA secondary structures based on the Watson-Crick base pairing rule. As A-U base pairs are less stable than G-C base pairs, miRNAs with no overlapping repeats are more stable than repeats-derived miRNAs with a larger A-U content and lower G-C content. Ho *et al.* have also reported that non-conserved pre-miRNAs in plants have a lower number of G-C pairings than conserved pre-miRNAs [23], which strongly supports the above observations about the extent of repetitive element-related miRNA conservation and G-C pairing content. This suggests that RrmiRNAs tend to be non-conserved or not well conserved in plants.

The sequence length, base composition and MFE are important features in predicting plant miRNAs, so this study investigated the MFE values of secondary structures for the two miRNA types. The MFE values were calculated using the RNAfold program [24]. Comparative analysis of MFE values revealed a significant difference between these two miRNA categories (*p*-value = 0.0012, Wilcoxon rank sum test) (Fig. 2C). The MFE values for RrmiRNAs (average −72.5 kcal mol^−1^) were significantly lower than those for NRrmiRNAs (average −63.6 kcal mol^−1^).

### Systematic analysis of tandemly duplicated miRNAs

**Figure 3:**
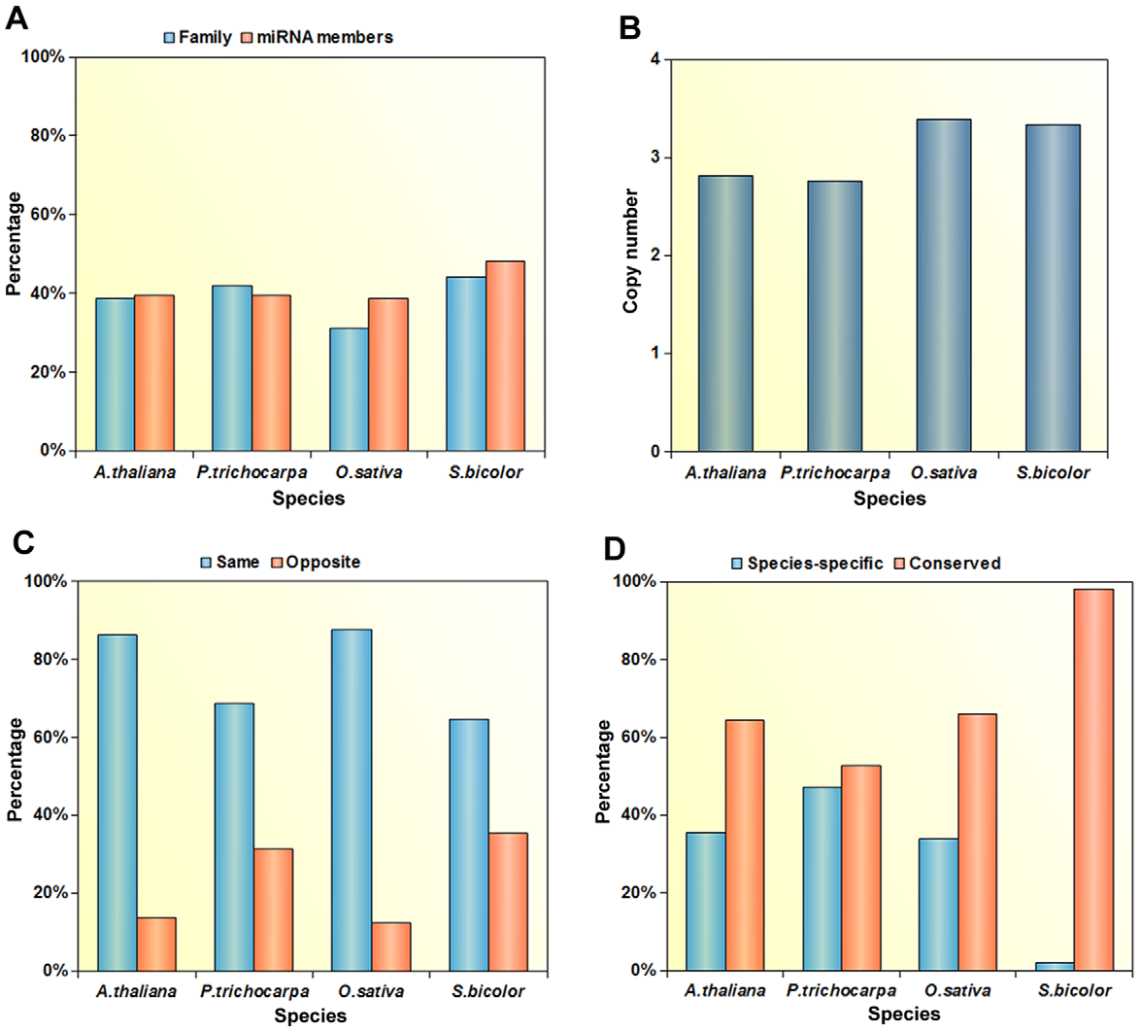
The overall percentage and copy number data for tandemly duplicated miRNAs in the four test plant species. (A) The percentage of miRNA families and miRNAs arising by tandem duplication with respect to the total number of miRNA families containing at least one miRNA, and the number of members of the corresponding miRNA family, respectively, for each species tested. (B) The average miRNA copy number in tandemly duplicated regions. (C) The percentage distribution of tandemly duplicated miRNAs on the same or opposite strands. (D) The percentage of conserved or species-specific tandemly duplicated miRNAs with respect to the total number of observed tandemly duplicated miRNAs for each species tested.

Previous studies in *A. thaliana* and *O. sativa* have suggested that tandem duplications often lead to closely related miRNAs that are located physically close to one another and generate nearby miRNA gene copies belonging to the same family [9], [18], [25]. To examine how important the role of tandem duplications is in miRNA family expansion in plants, this study mapped miRNA genes onto the corresponding genome and further examined the physical locations of all the members of various miRNA families. The miRNAs that had expanded through tandem duplications were identified as described in the Materials and Methods. Altogether, 248 miRNAs were found (45 in *A. thaliana*, 58 in *P. trichocarpa,* 95 in *O. sativa* and 50 in *S. bicolor*) from 51 miRNA families that had arisen by tandem duplications, which accounted for 40.7% of all miRNA family members and 37.8% of all miRNA families containing more than one miRNA (Fig. 3A and File S2). This suggested that tandem duplication events play an important role in increasing the production of novel miRNAs in plants and expanding miRNA families. When the miRNA copy number in tandemly duplicated regions was examined, it was found that the copy number of miRNAs in tandemly duplicated regions of eudicots (average 2.8) was lower than that for monocots (average 3.4) (Fig. 3B), implying that miRNAs in monocots might have experienced more tandem duplication events than in eudicots. The longest run of miRNAs derived from tandem duplication was sixteen (miR2118a∼2118a), which were continuously distributed in an 18 kb chromosomal region in *O. sativa*.

Previous studies have revealed that the same–strand miRNAs located in close genomic proximity to each other were co-transcribed as polycistronic units [25], [26], [27]. So a further investigation examined the plus/minus strand distribution for tandemly duplicated miRNAs (Fig. 3C). As shown in Fig. 3C, an average of 76.8% of tandemly duplicated miRNAs occurred on the same strand and are, therefore, more likely to be co-transcribed as polycistronic units and have similar expression levels. Tandemly duplicated miRNAs clustered on the opposite strand are more likely to be expressed at different levels, even within the same miRNA family, a result that had been observed for some family members in *Arabidopsis* and rice in a previous study [20]. Conservation comparisons between tandemly duplicated miRNAs in the four test plant species revealed that the percentage of conserved tandemly duplicated miRNAs varied from 98.0% in *S.bicolor* to 52.8% in *P. trichocarpa* which is relatively higher than the percentage of species-specific tandemly duplicated miRNAs in the corresponding species, implying that the tandem duplications may be more conserved during evolution in plants (Fig. 3D). These results also suggested that an important fraction of tandemly duplicated miRNAs seem to be products of recent tandem duplication events, and species-specific tandemly duplicated miRNAs may be derived from species-specific tandem duplication events and play specialized roles in different plant species.

### Genome-wide identification of miRNAs in duplicated blocks

**Figure 4:**
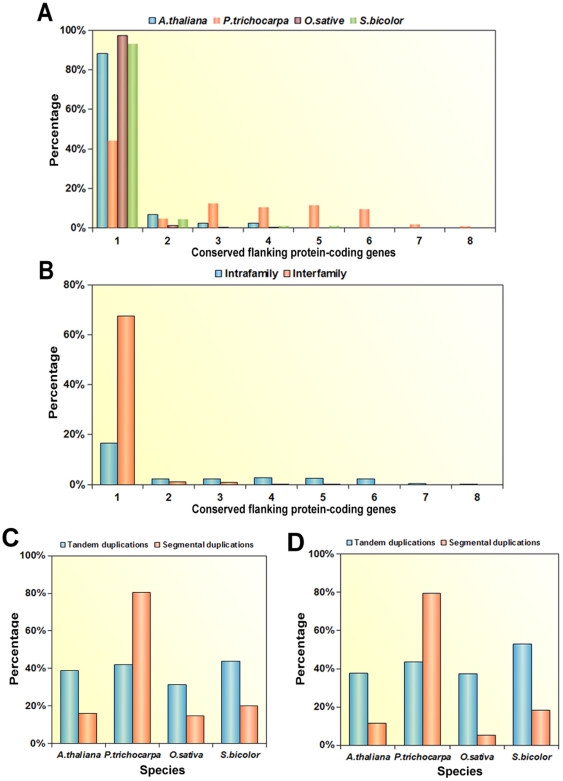
Characterization of miRNA duplicated blocks in the four test plant species. (A) The percentage of observed potentially duplicated blocks with different numbers of flanking conserved protein-coding genes with respect to the total number of potentially duplicated blocks for each test species. (B) Percentage comparison of observed potentially duplicated blocks with differing numbers of flanking conserved protein-coding genes with respect to the total number of potential duplicated blocks for inter-family and intra-family miRNAs. (C) Percentage comparison of miRNA families evolved from tandem and segmental duplications with respect to their respective totals. (D) Percentage comparison of miRNA family members evolved from tandem and segmental duplications with respect to their respective totals.

It is well known that tandem duplications and segmental duplications contribute to the origins and formation of miRNAs in animals [8], [10], [28]. Recent studies have reported that many miRNAs evolved from inverted duplication and local duplication in plants [9], [11], [27]. However, the origins and expansion of miRNA genes and miRNA families through segmental duplications are still poorly understood. To characterize the miRNAs that originated or belong to families that have been expanded by segmental duplication events in plants, perl scripts were used to extract 10 protein-coding genes that flanked the regions of each single miRNA or tandemly duplicated miRNAs. Then, the protein-coding genes flanking each miRNA were aligned using standalone BLAST. If one or more of the 10 protein-coding genes flanking the miRNA were found to have a best non-self match to protein-coding genes flanking another miRNA, as described in the Methods, these two miRNAs were considered as being possibly located in a duplicated block and perhaps originated from or had their families expanded by segmental duplication events. In this study, miRNA families containing a single gene were excluded and tandemly duplicated miRNAs were considered as single chromosomal loci. The results are summarized in Fig. 4 and File S3. A total of 462 duplicated chromosomal regions containing miRNAs (43 in *A. thaliana*, 100 in *P. trichocarpa,* 231 in *O. sativa* and 88 in *S. bicolor*) were identified as possible duplicated blocks in the four test plant species. Most duplicated blocks (88.4% in *A. thaliana*, 46% in *P. trichocarpa*, 97% in *O. sativa* and 93.2% in *S. bicolor*) had only one conserved flanking protein-coding gene. However, duplicated blocks in *P. trichocarpa* tended to contain more conserved flanking protein-coding genes than those in the other three test plant species. Furthermore, miRNAs in duplicated blocks were found to come either from the same family or from a different family. So the number of conserved protein-coding genes surrounding miRNAs within the same family (intra-family), and miRNAs from a different family (inter-family) (Fig. 4B) were compared. The results show that there were 133 duplicated blocks containing miRNAs within the same family that have at least one flanking protein-coding gene surrounding miRNAs, which accounted for only 28.8% of all duplicated blocks. Moreover, duplicated blocks containing miRNAs from different families tended to have fewer conserved protein-coding genes than the intra-family duplicated blocks. The 315 inter-family duplicated blocks, covering 68.2 % of all duplicated blocks, had only one flanking conserved protein-coding gene surrounding the miRNAs as opposed to 77 (16.7%) for the intra-family miRNA duplicated blocks. Therefore, to prevent overestimation of the number of duplicated blocks and make our analysis more stringent, only miRNAs located in duplicated blocks containing two or more conserved flanking protein-coding genes were considered as miRNAs derived from segmental duplications according to a previous study [9]. The number of segmental duplications and the number of flanking conserved protein-coding genes within each block are summarized in Table 1 and File S3 according to the above criteria. Within the four test plant species there are 59 intra-family duplication blocks that have more than one conserved flanking protein-coding gene and 11 inter-family duplication blocks. Also, there are more duplicated blocks and more flanking conserved protein-coding genes in *P. trichocarpa* compared with the other three test plant species (Table 2), suggesting that miRNAs in the perennial tree species tested appeared to experience more segmental duplication events, and were found in larger chromosomal regions, than in the other test plant species.

**Table 1:** The number of segmental duplications and flanking conserved protein-coding genes within each block.

Flanking conserved genes	Observed intra-family duplications a				Percent of intra-family duplications b				Observed inter-family duplications a				Percent of inter-family duplications b			
	ath	ptc	osa	sbi	ath	ptc	osa	sbi	ath	ptc	osa	sbi	ath	ptc	osa	sbi
2	2	3	1	4	2.86	4.29	1.43	5.71	1	2	2	0	1.43	2.86	2.86	0
3	1	9	1	0	1.43	12.86	1.43	0	0	4	0	0	0	5.71	0	0
4	1	10	1	1	1.43	14.29	1.43	1.43	0	1	0	0	0	1.43	0	0
5	0	11	0	1	0	15.71	0	1.43	0	1	0	0	0	1.43	0	0
6	0	10	0	0	0	14.29	0	0	0	0	0	0	0	0	0	0
7	0	2	0	0	0	2.86	0	0	0	0	0	0	0	0	0	0
8	0	1	0	0	0	1.43	0	0	0	0	0	0	0	0	0	0
Total	4	46	3	6	5.71	65.71	4.29	8.57	1	8	2	0	1.43	11.43	2.86	0

a The number of observed duplicated blocks, containing miRNAs within the same family or within the different family, that have at least two flanking protein-coding gene surrounding miRNAs.

b The percentage of observed duplicated blocks in each test species with respect to the total number of duplicated blocks.

ath: *A. thaliana*; ptc: *P. trichocarpa*; osa: *O. sativa*; sbi: *S. Bicolor.*

**Table 2:** The overview for origins and expansion of miRNAs derived from duplicated events in the four test plant species.

Species	Family	miRNAs in family	Family in tandem duplications	miRNAs in tandem duplications	Family in segmental duplications	miRNAs in segmental duplications
Ath	31	114	12	43	5	13
Ptc	31	147	13	64	25	117
Osa	48	246	15	92	7	13
Sbi	25	104	11	55	5	19

ath: *A. thaliana*; ptc: *P. trichocarpa*; osa: *O. sativa*; sbi: *S. bicolor*.

The study then examined whether the observed miRNA duplicated blocks were species-specific or conserved among the four test plant species. The results of conservation analysis for miRNA duplicated blocks in the four test plant species are summarized in Table 3. They show that observed intra-family duplications tended to be conserved and there were no species-specific intra-family duplicated blocks found in *Arabidopsis*, rice and sorghum suggesting that miRNAs located in these conserved intra-family duplicated blocks may have been subject to functional constraints that kept them highly conserved across the three species. However, in the *P. trichocarpa* genome, 31 of the 46 (67.4%) intra-family duplicated blocks were species-specific. In contrast, all the observed inter-family duplications were species-specific and there was no conservation across the four test plant species. Furthermore, there were no common duplicated blocks of miRNAs in the four test plant species, either within the same family or between different families.

**Table 3:** The results of conservation analysis for miRNA duplicated blocks in the four test plant species.

Species	Observed intra-family duplications					Observed inter-family duplications				
	Total	Species- specific	Conserved in two species	Conserved in three species	Common	Total	Species- specific	Conserved in two species	Conserved in three species	Common
Ath	4	0	4	0	0	1	1	0	0	0
ptc	46	31	12	3	0	8	8	0	0	0
osa	3	0	1	2	0	2	2	0	0	0
sbi	6	0	3	3	0	0	0	0	0	0

ath: *A. thaliana*; ptc: *P. trichocarpa*; osa: *O. sativa*; sbi: *S. bicolor*.

To provide a further overview on the origins and family expansion of miRNAs derived from duplication events in the four test plant species studied, the number of tandem and segmental duplications for each miRNA family, containing more than a single miRNA, were counted and the results are shown in Table 2. Taken together, out of the four test species, 76 out of 135 (56.3%) miRNA families (15 in *A. thaliana*, 26 in *P. trichocarpa*, 21 in *O. sativa* and 14 in *S. bicolor*) arose by either tandem or segmental duplication events, or a combination of the two. As shown in Table 2, 51 miRNA families were found to be involved in tandem duplications, and 42 miRNA families are involved in segmental duplications. Only 17 miRNA families evolved by a combination of tandem and segmental duplication events in the four test plant species studied. Of these families, 254 (41.6%) miRNAs were involved in tandem duplications and 162 (26.5%) miRNAs were involved in segmental duplications. To determine the relative importance of tandem and segmental duplications in the evolution and expansion of miRNA families in the four test plant species, a comparative analysis of families and miRNAs evolved from tandem and segmental duplications among four different plant species was performed (Fig. 4 C, D). In total, 12 *Arabidopsis*, 15 rice and 11 sorghum miRNA families arose by tandem duplications, corresponding to 38.7%, 31.3% and 44% of their respective overall miRNA totals. These values were much higher than the percentage of miRNA families that arose by segmental duplication in *Arabidopsis* (16.1%), rice (14.6%) and in sorghum (20%). However, a relatively higher proportion of miRNA families underwent segmental duplications (80.7%) than underwent tandem duplications (41.9%) in *P. trichocarpa*. This trend was similar for miRNAs originating from tandem and segmental duplications among the four test plant species. These results revealed the differences in the relative importance of tandem and segmental duplications to the origins and expansion of miRNA families in the four test plant species.

## Discussion

Although miRNAs derived from repetitive elements and miRNA families that expanded through genomic duplication events have been well characterized in animals [6], [8], [10], [28], [29], the characterization of repetitive element-related miRNAs and the expansion of miRNAs and miRNA families through genomic duplication events in plants remain largely unknown. Several studies have focused on TE-derived miRNAs in *A. thaliana* and *O. sativa* [7], [16]. In this study, a more extensive analysis was undertaken to provide a complete mapping of all the miRNAs on repetitive elements in four plant species at the genome scale. A total of 163 miRNAs evolving from repetitive elements (RrmiRNAs) were identified in the four test plant species that had at least one high-scoring sequence pair with repetitive sequences. These results provide additional evidence, and strongly support the idea that small RNA fragments that map to genome regions annotated as repetitive elements should not be discarded [8]. Moreover, this study also analyzed the genomic distribution and evolutionary patterns of repetitive element-related miRNAs. These results suggested that the repetitive element-related miRNAs showed significantly different distributions in intragenic or intergenic regions compared to all miRNAs present in the species tested. Repetitive element-related miRNAs were also more likely to arise from intragenic regions in *O. sativa* and *S. bicolor* than they would from intragenic regions in *A. thaliana* and *P. trichocarpa*. Only 11% of miRNAs that overlapped repetitive elements were highly conserved in plants, and most were species-specific, which is similar to recent findings for repetitive related-miRNAs in animals [8]. This suggested that these repeats-related miRNAs could be evolutionary young miRNAs, which are possibly involved in species-specific regulatory effects as reported in Fahlgren's study [30].

Through above results, it was observed that RrmiRNAs and NRrmiRNAs may have different origins and evolutionary pathways. Therefore, it is proposed that expressional and functional diversification may exist between RrmiRNAs and NRrmiRNAs. Further analysis was undertaken comparing various sequence and structural characteristics of repetitive element-related miRNAs to that of non-repeat-derived miRNAs, including hairpin precursor sequence length, base composition and MFEs. A number of differing characteristics were observed between RrmiRNAs and NRrmiRNAs: (1) RrmiRNAs had longer hairpin precursor sequences than NRrmiRNAs; (2) the G-C content in hairpin precursor sequences of RrmiRNAs was lower than that in NRrmiRNAs and (3) RrmiRNAs had relatively lower MFEs than NRrmiRNAs. In order to examine whether these differences were statistically significant or not, Wilcoxon rank sum test analysis was performed. The analysis indicated that these differences were statistically significant. These differences in hairpin precursor sequences and structural features may reflect an evolutionary and biogenesis pathway divergence between RrmiRNAs and NRrmiRNAs, which suggests that there may also be expressional and functional diversification between them. In plants, miRNAs derived from repetitive elements tend to form less stable secondary structures and have a low level of expression. In animals, several studies have shown that new miRNA genes are transcribed at a lower rate compared with older ones [31], [32]. Recent studies on rice and maize have found that a subset of lineage-specific families, co-located with TE sequences, were usually weakly expressed [19], [22], which is consistent with observations from this study. Moreover, these significant differences between RrmiRNAs and NRrmiRNAs might promote further study into miRNAs identification in plants.

In order to gain new insights into miRNAs that have originated or have had families expanded by tandem or segmental duplication events in plants, a systematic study, using a combination of computational and comparative genomic approaches, was undertaken to identify and characterize the miRNAs that have originated or have had families expanded by tandem or segmental duplication events in the four test plants. This expanded Maher's results, which concentrated on only a very limited number of miRNA genes in *A. thaliana* [9], to all currently known miRNA genes in the four test plant species and further investigated the evolutionary patterns of miRNAs associated with duplication events. The results of this analysis showed that tandem duplication events have played a significant role in the origins and family expansion of miRNAs in plants. A considerable number of miRNAs were observed as tandemly duplicated miRNAs. Furthermore, this study found that the copy number and conservation of miRNAs in tandemly duplicated regions varied between plant species. It has been reported that the duplicated copies of protein-coding genes might take on new expression patterns and result in sub-functionalization and neo-functionalization [18], [33]. So, it is reasonable to believe that some miRNAs derived from tandem duplication events might acquire novel promoters and diverged sequences, which provide opportunities to generate novel spatial and temporal regulatory patterns and thus increase the complexity of the miRNA regulatory network. This was observed in a previous study on tandem duplicated paralogs of miR395 in rice and miR168 in Brassicaceae [34], [35]. In addition, most of the tandemly duplicated miRNAs in this study were found to be located on the same strand and were therefore more likely to be co-transcribed as polycistronic units, suggesting that in plants, miRNA copy number changes in tandemly duplicated regions would have a dosage effect on a given miRNA from a single promoter. This has been reported in the previous analysis performed by Merchan *et al.* [36].

To characterize the miRNAs originating or having families expanded by segmental duplication events in plants, all miRNA families containing more than one miRNAs in the four test plant species were analyzed, and 59 intra-family duplication blocks and 11 inter-family duplication blocks were found. These results provided additional evidence, and strongly suggested that duplication events (tandem and segmental) promoted the expansion of miRNAs families in plants. However, comparative analysis on the relative importance of tandem and segmental duplications in the evolution and expansion of miRNA families suggested that the contribution made by these two duplication events differed greatly between the test perennial tree species and the other three annual species. The expansion of miRNA families originating from tandem duplication events in *Arabidopsis*, rice and sorghum was more likely than miRNA families expanding through segmental duplications. In contrast, in *P. trichocarpa*, genomic segmental duplications contributed significantly to the expansion of miRNA families. Barakat *et al.* analyzed the number of members per miRNA family and showed that most families expanded in size to a greater extent in *Populus* than they did in *Arabidopsis* and rice [37]. The results from this study, taken together, suggest that the divergence in miRNA family sizes between *Arabidopsis*, rice, sorghum and *P. trichocarpa* may be due to the differences in the relative importance of tandem and segmental duplications in the expansion of miRNA families in the four test plant species. Further analysis showed that most intra-family duplicated blocks in *P. trichocarpa* were species-specific suggesting that miRNAs located in these duplication blocks undergo *P. trichocarpa*-specific expansion resulting in the increasing number of miRNA members within a family. Other studies have shown that larger miRNA families in *Populus* appear to be advantageous for perennial growth and for adaptation to different ecological environments [37].

In conclusion, based upon bioinformatics analysis of miRNAs and repetitive elements, this study identified and characterized the repetitive element-related miRNAs of four test plant species and carried out a series of studies to compare the characteristics of repetitive element-related miRNAs to that of non-repeat-derived miRNAs. This study has demonstrated that repetitive element-related miRNAs and non-repeat-derived miRNAs showed significantly different characteristics, including their location in protein-coding and intergenic regions in genomes, their conservation in the four test plant species, sequence length of their hairpin precursor, base composition of their hairpin precursor and the minimum free energy of their hairpin structures. Furthermore, a combination of computational and comparative genomic approaches was used to systematically identify and characterize the miRNAs that had originated from, or had families that had been expanded by, tandem or segmental duplication events in the four test plants. These results show the important roles tandem or segmental duplication events have played in the origins and expansion of miRNAs in the annual plants and the perennial plant species tested. Because this study was based on a computational approach and the possibility of incomplete identification of plant miRNAs exists, these results may contain a certain number of false positives. However, the overall results suggest that this study characterized miRNAs derived from repetitive elements and duplication events with a high degree of accuracy at the genome scale and provides new knowledge and a deeper insight into the origins and evolution of miRNAs in plants.

## Materials and Methods

### Data sets

This analysis tested four plant species: *Arabidopsis thaliana*, *Populus trichocarpa*, *Oryza sativa* and *Sorghum bicolor* because the number of miRNA genes in these four species is relatively large and the genome sequences and annotations for the four species are relatively complete. All the known miRNA foldback sequences and genome coordinates in the four plant species were retrieved from the miRBase Sequence Database, release 17 (http://www.mirbase.org/) [38]. Currently, the miRBase database lists 232 miRNAs in *A. thaliana*, 234 miRNAs in *P. trichocarpa*, 491 miRNAs in *O. sativa* and 148 miRNAs in *S. bicolor*. Those miRNAs that were not mapped to corresponding genomes were discarded. This left 231 (*A. thaliana*), 190 (*P. trichocarpa*), 456 (*O. sativa*) and 148 (*S. bicolor*) genome-mapped miRNAs in this study. The genome assembly annotation and protein-coding genes for *A. thaliana* (TAIR 9) were downloaded from the Arabidopsis Information Resource (TAIR, http://www.arabidopsis.org) [39]. The genome assembly annotation and protein-coding genes for *O. sativa* (MSU6.0) were downloaded from the Rice Genome Annotation Project (http://rice.plantbiology.msu.edu/) [40]. The genome assembly annotation and protein-coding genes for *P. trichocarpa* (JGI_Poptr2.0) and *S. bicolor* (JGI_sbi1) were downloaded from Phytozome v7.0 (http://www.phytozome.net/) [15], [41].

### Genome-wide identification of repeats-related plant miRNAs

The repetitive DNA sequences of the three annual plant species (*A. thaliana*, *O. sativa* and *S. bicolor*) were retrieved from the Plant Repeat Database (http://plantrepeats.plantbiology.msu.edu/) [42]. The repetitive elements in *P. trichocarpa* genome were taken from the RepPop database (http://csbl.bmb.uga.edu/~ffzhou/RepPop) [13]. Hairpin precursor sequences of miRNAs were aligned using standalone BLAST (blastn, version 2.2.27) against repetitive sequences of corresponding species. The miRNAs were considered as repetitive element-related miRNAs (RrmiRNAs) when they exhibited at least one high-scoring sequence pair with repetitive sequences according to the criteria reported by Li *et al.* in their latest study [7]. The miRNAs with no overlapping repetitive sequences were defined as non-repeat-related miRNAs (NRrmiRNAs).

### Systematic analysis of tandemly duplicated miRNAs

The known miRNA families were based on the definitions from the miRBase [38]. The miRNA genes were mapped onto the corresponding genome and a search for further physical locations was undertaken for all the members in the different miRNA families. If multiple miRNA members within a miRNA family were distributed in the same chromosome contiguously and retained their physical linkage over long evolutionary timescales without regard to whether they were on the same or opposite strands, then these miRNAs were assigned as tandemly duplicated miRNAs according to criteria used in previous studies [9], [18], [25].

### Genome-wide identification of miRNAs in duplicated blocks

In order to determine whether the miRNAs arose or evolved from segmental duplication events, genome-wide analysis was undertaken to examine whether a miRNA resides within a duplicated block as previously described by Maher *et al.* [9]. First, perl scripts were used to extract 10 protein-coding genes flanking regions for each single miRNA or tandemly duplicated miRNAs. Then the protein-coding genes flanking each miRNA were aligned, using standalone BLAST (blastn, version 2.2.27), against those protein-coding genes surrounding another miRNA in order to identify paralogs. For each miRNA pair, the number of protein-coding genes with the best non-self match to protein-coding genes flanking another miRNA was counted.

### Evolutionary conservation analysis of miRNAs derived from repetitive sequences and duplication events

To better understand the degree of conservation of miRNAs derived from repetitive sequences and duplication events in the four test plant species, the homologous miRNAs were identified based on the reciprocal best BLAST hit (RBH) method. The RBH method was performed running a BLASTN search for miRNA hairpin precursor sequences from different plant species and selecting the highest bit score, always setting the lowest acceptable E-value limit to 1e-06 according to previous studies in plants [35], [43]. E-values, together with bit scores, were reported for the BLAST analysis, which prevents overestimation of plant miRNA homologs and makes the analysis more stringent. Then the miRNAs were divided into three groups according to their degree of evolutionary conservation, as described by Zhou *et al*. [27]. If homologues were simultaneously found in monocots and eudicots, the miRNAs were considered as highly conserved miRNAs. Those miRNAs found only in monocots or eudicots were considered as low conserved miRNAs, and those found only in one species were considered as species-specific miRNAs.

## Supporting Information

File S1 **Information of miRNAs that overlap with repetitive elements in the four test plant species.**
(XLS)

File S2 **Information of tandemly duplicated miRNAs in the four test plant species.**
(XLS)

File S3 **Information of flanking conserved protein-coding genes within each block in the four test plant species.**
(XLS)
